# Multiplex ligation-dependent probe amplification identifies copy number changes in normal and undetectable karyotype MDS patients

**DOI:** 10.1007/s00277-021-04550-8

**Published:** 2021-05-15

**Authors:** Jing Ma, Xiaofei Ai, Jinhuan Wang, Limin Xing, Chen Tian, Hongliang Yang, Yong Yu, Haifeng Zhao, Xiaofang Wang, Zhigang Zhao, Yafei Wang, Zeng Cao

**Affiliations:** 1 Department of Hematology and Blood and Marrow Transplantation, Tianjin Medical University Cancer Institute and Hospital, National Clinical Research Center for Cancer, Tianjin’s Clinical Research Center for Cancer, Key Laboratory of Cancer Prevention and Therapy, Huan-Hu-Xi Road, Ti-Yuan-Bei, Hexi District, Tianjin, 300060 China; 2 Department of Pathology, Institute of Hematology and Blood Diseases Hospital, Chinese Academy of Medical Sciences & Peking Union Medical College, No. 288 Nanjing Road, Heping District, Tianjin, 300020 China; 3 Department of Oncology, The Second Hospital of Tianjin Medical University, No.23 Pingjiang Road, Hexi District, Tianjin, 300211 China; 4 Hematology Department of General Hospital, Tianjin Medical University, No.154 Anshan Road, Heping District, Tianjin, 300052 China

**Keywords:** Myelodysplastic syndromes, Cytogenetic analysis, Multiplex ligation-dependent probe amplification; Normal karyotype, Undetectable chromosome pattern

## Abstract

Chromosomal abnormalities play an important role in classification and prognostication of myelodysplastic syndrome (MDS) patients. However, more than 50% of low-risk MDS patients harbor a normal karyotype. Recently, multiplex ligation-dependent probe amplification (MLPA) has emerged as an effective and robust method for the detection of cytogenetic aberrations in MDS patients. To characterize the subset of MDS with normal karyotype or failed chromosome banding analysis, we analyzed 144 patient samples with normal karyotype or undetectable through regular chromosome banding analysis, which were subjected to parallel comparison via fluorescence in situ hybridization (FISH) and MLPA. MLPA identifies copy number changes in 16.7% of 144 MDS patients, and we observed a significant difference in overall survival (OS) (median OS: undefined vs 27 months, p=0.0071) in patients with normal karyotype proved by MLPA versus aberrant karyotype cohort as determined by MLPA. Interestingly, patients with undetectable karyotype via regular chromosome banding indicated inferior outcome. Collectively, MDS patients with normal or undetectable karyotype via chromosome banding analysis can be further clarified by MLPA, providing more prognostic information that benefit for individualized therapy.

**Supplementary Information:**

The online version contains supplementary material available at 10.1007/s00277-021-04550-8.

## Introduction

Myelodysplastic syndrome (MDS) is a heterogeneous group of hematologic neoplasms classically described as a clonal disorder of hematopoietic stem cells leading to dysplasia and ineffective hematopoiesis in the bone marrow [1]. Chromosomal abnormalities play an important role in classification and prognostication of MDS patients; however, more than 50% of low-risk MDS patients harbor a normal karyotype as revealed by regular chromosome banding analysis [2, 3]. While chromosome banding analysis can only detect gains and/or losses of more than 10 Mb size, it depends on proliferation of the MDS clone to obtain metaphases in vitro. Therefore, it is necessary to find a more targeted, high-throughput, simple, and cost-effective method with higher resolution and accuracy targeting at the clinically relevant lesions which have been described in MDS patients.

Multiplex ligation-dependent probe amplification (MLPA) assay is a technique for copy number variation (CNV) identification in many human genes simultaneously. So far, over 300 probe sets specific for a very large range of genetic disorders are commercially available. MLPA is a multiplex polymerase chain reaction (PCR)-based technique that can quantify up to 50 different genomic targets simultaneously in a single experiment through amplification of specific hybridizing probes [4–7]. One of the major advantages is the high specificity, because it can distinguish sequences differing in length by only one nucleotide. Another advantage is the low amount of input DNA (minimum of 20–50 ng) required for a successful MLPA reaction [8]. In this study, we addressed the question whether MDS patients with normal or without result after banding cytogenetics harbors cytogenetically cryptic gains or losses could be detected by MLPA.

## Methods

### Patients and samples

A total of 258 patients from January 2008 to December 2018 were retrospectively enrolled and were diagnosed with MDS according to the World Health Organization (WHO) Criterion 2016. Bone marrow samples at initial diagnosis of these patients were obtained. Among these patients, 144/258 (55.8%) cases showed either normal karyotype (n=132; 51.2 %) or without result after banding cytogenetics (n=12; 4.6%). The male to female ratio was 1.48:1, and the median age of the patients was 53 years old (range: 15–83 years old) with the median follow-up time of 18 months (range: 2–101 months). Patients were given the informed consent to genetic analyses and laboratory data for research studies. The study was approved by the Medical Ethics Committee of the Tianjin Cancer Institute and Hospital.

### Cytogenetic analysis

Cytogenetic studies using standard G-banding techniques on heparinized BM samples were performed as part of the diagnostic work-up. At least 20 metaphase cells were analyzed whenever possible. Clonal abnormalities were defined as 2 or more cells with the same chromosomal gain or structural rearrangement or at least 3 cells with the same chromosomal deletion. Chromosome identification and karyotype descriptors used the International System for Human Cytogenetic Nomenclature (ISCN) [9].

### Interphase fluorescence in situ hybridization (FISH)

FISH was performed on whole bone marrow mononuclear cells. In brief, commercial, multicolor probes provided by Vysis laboratories (Abbott Laboratories, IL, USA) were used that included probes for -5/-5q, -7/-7q, +8, -20q, and 17p-, respectively. The following probes were used: LSI (locus-specific identifier) EGR1/D5S23, D5S721 Dual Color Probe for chromosome 5q; LSI D7S522/CEP 7 Probe for chromosome 7q; CEP (centromere enumeration probe) 8 DNA Probe for chromosome 8; LSI D20S108 Probe for chromosome 20q; and TP53 Probe for chromosome 17; all probes were obtained from Vysis Inc. (Downers Grove, IL, USA). The evaluation of FISH signals was performed using a fluorescence microscope (Olympus BX51, Japan) equipped with Q-FISH imaging software (IMSTAR, France). We counted 200–300 interphase nuclei for each slide. The positive threshold percentages were established according to previously published recommendations. The FISH results were interpreted in each institution by at least two experienced molecular pathologists, independent of concurrent metaphase karyotyping. Results were described according to the ISCN criteria [9].

### Multiplex ligation-dependent probe amplification

Bone marrow specimens were collected from patients at diagnosis. Genomic DNA was extracted using the AxyPrep Blood Genomic DNA Miniprep Kit (Axygen Biosciences, cat no. AP-MN-BL-GDNA-250 Union city, CA, USA). Fifty nanograms of gDNA were subjected to MLPA analysis by using SALSA MLPA P414-A1 MDS probe mix (MRC-Holland, Amsterdam, Netherlands). The probe mix contained 46 probes targeting at chromosomal regions of interest in MDS and 12 internal reference gene probes targeting at regions that are generally unchanged in MDS. MLPA reactions including internal quality controls and negative controls were performed according to the manufacturer instructions. The PCR products were analyzed using ABI 3130XL Genetic analyzer (Applied Biosystems, Foster City, CA, USA) and Coffalyser.net software (MRC Holland, Amsterdam, Netherlands) according to the manufacture instruction. In addition, 10 DNA samples derived from the peripheral blood of healthy donors were subjected to MLPA analysis. The “Mean±2SD” (95% CI, P=0.05) and “Mean±3SD” (95% CI, P=0.01) values for each individual probe are listed in our raw data. To improve the evaluation of the results with a larger CI, the “Mean±3SD” reference range was used as the cutoff value for CNV determination in our study.

### Statistical analysis

Survival curves were plotted by the Kaplan-Meier method, and the difference was assessed by log-rank test. Overall survival (OS) was measured from the time of diagnosis to the date of death or last follow-up. The statistically significant difference was considered at p < 0.05.

## Results

### Characterization of patients with normal karyotype or no result after banding cytogenetics

**Fig. 1:**
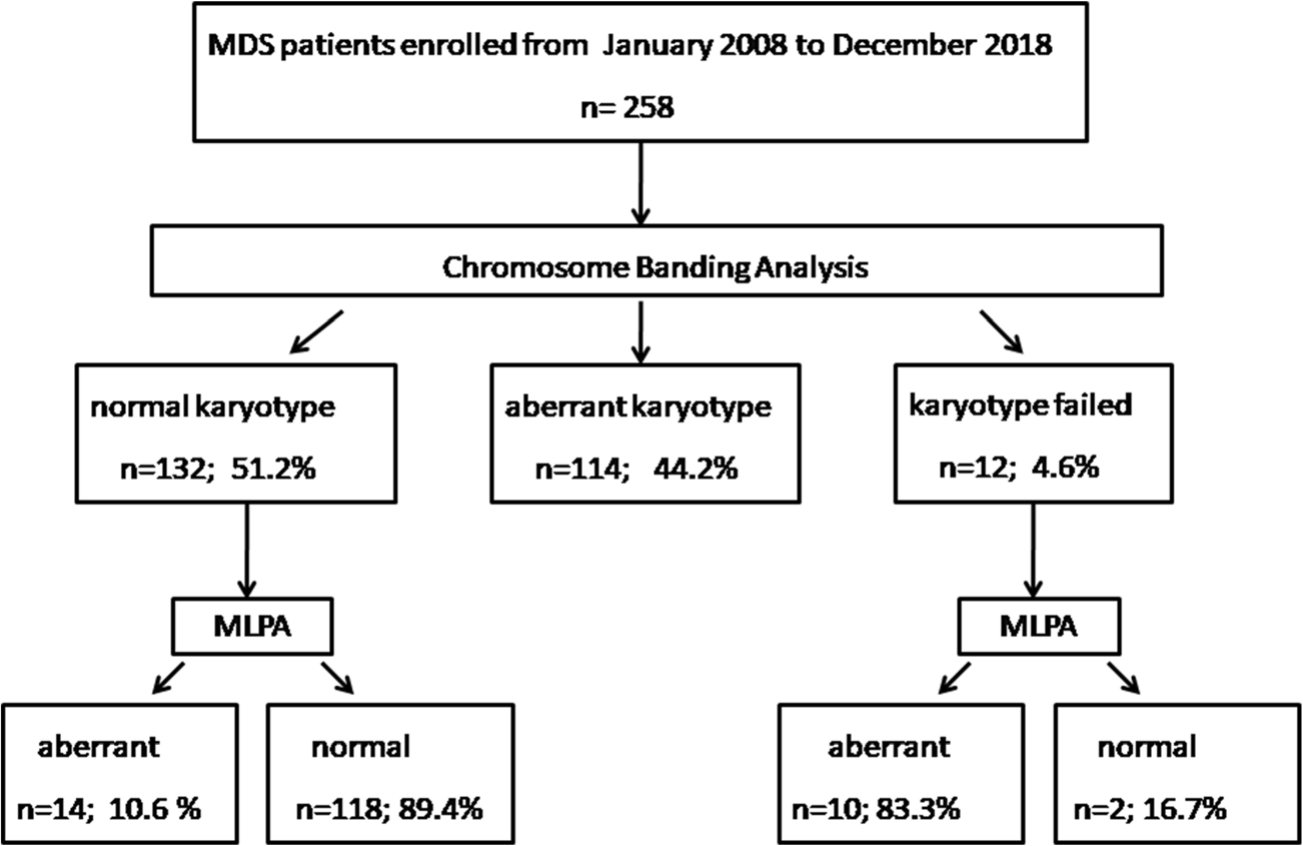
Diagnostic approach in 258 MDS patients. Distribution of patients with aberrant karyotype, normal cytogenetics, and failed chromosome banding analysis is depicted

As shown in Fig. 1, normal karyotype and cases without result after banding cytogenetics are approximately 55.8%. MLPA identifies copy number changes in 24 (16.7%, 24/144 ) patients. Among these 24 patients, 10 patients showed chromosome banding analysis failed. For patients with normal karyotype, 10.6% (14/132) were identified with CNVs. Characteristics of 144 patients are shown in Table 1. The 144 MDS patients were divided into four subgroups based on MLPA and karyotype results; there were 86 males and 58 females. According to the classification of WHO 2016 version, the most common subtype is MDS-MLD. We calculated the Revised International Prognostic Scoring System scores (IPSS-R) and confirmed that 8 patients were very high risk, 28 patients with high risk, 48 patients with intermediate risk, 50 patients with low risk, and 10 patients with very low-risk disease.

**Table 1:** Characteristics of MDS patients in our study (n=144)

	Total n (%)	Normal karyotype and MLPA+	Normal karyotype and MLPA−	Failed karyotype and MLPA+	Failed karyotype and MLPA−
N. of patients	144 (100%)	14	118	10	2
Median age (years)	53	47.5	57	44	51
Sex					
Male	86(59.7%)	13	66	6	1
Median Hgb (g/L)	76	72	81	76	79
Median ANC (×10^9^/L)	1.2	1.0	1.1	0.9	1.2
Median platelet count (×10^9^/L)	72	89	76	64	70
WHO 2016					
MDS-SLD	1 (0.7%)	0	1	0	0
MDS-RS	6 (4.2%)	1	4	1	0
MDS-MLD	68 (47.2%)	6	56	4	2
MDS-EB-1	33 (22.9%)	4	27	2	0
MDS-EB-2	29 (20.1%)	3	25	1	0
MDS-U	7 (4.9%)	0	5	2	0
IPSS-R risk category					
Very low	10 (7.0%)	2	8	0	0
Low	50 (34.7%)	6	42	2	0
Intermediate	48 (33.3%)	1	46	1	0
High	28 (19.4%)	4	16	6	2
Very high	8 (5.6%)	1	6	1	0

### Abnormalities detected by MLPA in 14 MDS patients with normal karyotype

Among 24 patients, 14 patients showed normal karyotype, which are shown in Table 2. According to cytomorphology, the cohort comprised the following MDS subtypes: MDS-RS (n=1), MDS-MLD (n=6), MDS-EB-1 (n=4), and MDS-EB-2 (n=3). The most common CNV was -17p (P53-4b, TP53-1).

**Table 2:** Copy number changes identified by MLPA in 14 MDS patients with normal karyotype

Case	Age	Gender	Diagnosis	Karyotype based on MLPA	Assumed karyotype according to MLPA
Patient 1	69	Male	MDS-EB-1	11q(4): KMT2A-4,KMT2A-36,TIRAP-3,ETS1-10	+11q
Patient 2	72	Male	MDS-RS	11q(4): KMT2A-4 ,KMT2A-36,TIRAP-3,ETS1-10	+11q
Patient 3	50	Male	MDS-MLD	20q(1): ASXL1-4	-20q
Patient 4	63	Male	MDS-EB-2	8q(3): NCOA2-5,MYC-3,PTK2-33 17p(2): TP53-4b,TP53-1	+8q -17p
Patient 9	39	Male	MDS-MLD	17p(2): TP53-4b,TP53-1	-17p
Patient 10	36	Male	MDS-MLD	17p(3): TP53-10,TP53-4b,TP53-1	-17p
Patient 11	46	Male	MDS-EB-2	17p(2): TP53-4b,TP53-1	-17p
Patient 12	51	Male	MDS-MLD	11q(1): KMT2A-4(+) 17q(1): NF1-17 SUZ12-12(+)	-17p
Patient 13	42	Male	MDS-EB-1	17q(1): NF1-17	-17q
Patient 14	34	Female	MDS-EB-2	19p(1):SMARCA4-25 19q(1): PRPF31-14	-19
Patient 15	32	Male	MDS-MLD	20q(2):MMP9-9,ZMYND8-14	-20q
Patient 16	57	Male	MDS-EB-1	20q(3): ASXL1-4,SRC-6,ZMYND8-14	-20q
Patient 19	47	Female	MDS-MLD	5q(3):APC-18,EGR1-1,EGR1-2	-5q
Patient 24	61	Female	MDS-EB-1	7q(4): CDK6-8 SAMD9L-5,MLL5-4,MET-13	-7q

Chromosome 8 abnormality was positive in 1 case (7.1%, 1/14); it showed 8q amplification. Chromosome 5 abnormality was positive in 1case (7.1%, 1/14). Chromosome 7 abnormality was positive in 1 case (7.1%, 1/14). Chromosome 20 abnormalities were positive in 3 cases (21.3%, 3/14). Chromosome 17 abnormalities including both17p and 17q deletions were positive in 6 cases (42.9%, 6/14), 5 patients for 17p deletion, and 1 patient for 17q deletion. Chromosome 11 abnormalities were positive in 2 cases (14.2%, 2/14), and both showed 11q amplifications. One patient showed chromosome19 abnormalities including both 19p and 19q deletions. All detected aberrations are summarized in Table 2.

### Abnormalities detected by MLPA in 10 MDS patients without result after banding cytogenetics

Among 24 patients, 10 patients with no result after banding cytogenetics are shown in Table 3. The cohort involved the following MDS subtypes: MDS-RS (n=1), MDS –MLD (n=4), MDS-EB-1 (n=2), MDS-EB-2 (n=1), and MDS-U(n=2). The most common CNVs were −7 and +8.

**Table 3:** Copy number changes identified by MLPA in 10 MDS patients with failed chromosome banding analysis

Case	Age	Gender	Diagnosis	Karyotype based on MLPA	Assumed karyotype according to MLPA
Patient 5	81	Male	MDS-EB-1	8p(1): FGFR1-2 8q(4): NCOA2-5,RUNX1T1-8, MYC-3, PTK2-33	+8
Patient 6	49	Male	MDS-MLD	8p(1): FGFR1-2 8q(4): NCOA2-5,RUNX1T1-8,MYC-3, PTK2-33	+8
Patient 7	50	Male	MDS-EB-1	8p(1): FGFR1-2 8q(4): NCOA2-5, RUNX1T1-8,MYC-3,PTK2-33	+8
Patient 8	42	Male	MDS-MLD	8p(1): FGFR1-2 8q(4): NCOA2-5, RUNX1T1-8,MYC-3,PTK2-33 11q(1): KMT2A-4	+8 +11q
Patient 17	39	Female	MDS-U	5q(3): EGR1-1,EGR1-2,RPS14-3 17p(3):TP53-10,TP53-4b,TP53-1 19p(1):SMARCA4-25 19q(1): PRPF31-14	-5q -17p -19
Patient 18	48	Male	MDS-MLD	5q(4):EGR1-1,MIR145-1,SPARC-7,SPARC-1	-5q
Patient 20	37	Female	MDS-U	7p(1): IKZF1-30 7q(7):CDK6-8,SAMD9L-5,EPO-4,MLL5-4 ,MET-13,EZH2-20,EZH2-13	-7
Patient 21	41	Male	MDS-MLD	7p(1): IKZF1-30 7q(7): CDK6-8,SAMD9L-5,EPO-4,MLL5-4 ,MET-13,EZH2-20,EZH2-13	-7
Patient 22	41	Male	MDS-EB-2	7q(3): CDK6-8,MLL5-4,MET-13	-7q
Patient 23	67	Male	MDS-RS	7q(5): CDK6-8,MLL5-4,MET-13,EZH2-20,EZH2-13	-7q

Chromosome 8 abnormalities including both 8p and 8q amplifications were positive in 4 cases (40%, 1/10). Chromosome 5 abnormalities were positive in 2 cases (20%, 2/10). Chromosome 7 abnormalities including both 7q deletion and 7p deletion were positive in 4 cases (40%, 4/10), with two patients including both 7p and 7q deletions. Chromosome 20 abnormalities were not detected. Chromosome 17 abnormality was positive in 1 case (10%, 1/10). Chromosome11 abnormality was positive in 1 case (10%, 1/10). One patient showed chromosome 19 abnormalities including both 19p and 19q deletions. All detected aberrations are summarized in Table 3.

### Comparison of MLPA assay and FISH

To evaluate the performance of MLPA as a candidate method for the identification of CNVs in MDS patients, five abnormalities, including -5/-5q, -7/-7q, +8, -20q, and 17p-, were detected by FISH and MLPA. FISH results of 144 cases were compared with that of MLPA. Among 144 MDS patients, 137 results were concordant, and the whole consistency was 95.1%. The genetic lesions determined by FISH and MLPA are listed in Table 4. Using MLPA analysis, clonal cytogenetic abnormalities were detected in 24 MDS patients with normal and undetectable karyotype, and 19/24 (79.2%) of those patients were reclassified into a higher-risk IPSS-R prognostic category. Using FISH, 62.5% (15/24) of MDS patients showed chromosomal abnormalities, whereas MLPA analysis showed that 100% (24/24) of MDS cases contained at least one CNV. Patient 8 and patient 17 showed two CNVs and three CNVs of MLPA analysis, respectively. All the additional detected aberrations by MLPA are summarized in Table 4.

**Table 4:** Genetic lesions determined by FISH and MLPA

Case	Diagnosis	IPSS-R risk	Karyotype	FISH	MLPA	IPSS-R risk group by MLPA
Patient 5	MDS-EB-1	Very high	Failed	+8	+8	Very high
Patient 6	MDS-MLD	High	Failed	+8	+8	Very high
Patient 7	MDS-EB-1	High	Failed	+8	+8	Very high
Patient 8	MDS-MLD	High	Failed	+8	+8 +11q	Very high
Patient 18	MDS-MLD	High	Failed	-5q	-5q	High
Patient 20	MDS-U	Intermediate	Failed	-7	-7	Very high
Patient 22	MDS-EB-2	High	Failed	-7q	-7q	Very high
Patient 23	MDS-RS	Low	Failed	-7q	-7q	Very high
Patient 17	MDS-U	Intermediate	Failed	-5q	-5q -17p -19	Very high
Patient 4	MDS-EB-2	Very high	Normal	+8 -17p	+8 -17p	Very high
Patient 9	MDS-MLD	Low	Normal	-17p	-17p	High
Patient 10	MDS-MLD	Low	Normal	-17p	-17p	High
Patient 15	MDS-MLD	Low	Normal	-20q	-20q	Low
Patient 16	MDS-EB-1	Low	Normal	-20q	-20q	Intermediate
Patient 24	MDS-EB-1	High	Normal	-7q	-7q	Very high
Patient 21	MDS-MLD	High	Failed	Negative*	-7	Very high
Patient 1	MDS-EB-1	High	Normal	Negative	+11q	High
Patient 2	MDS-RS	High	Normal	Negative	+11q	High
Patient 3	MDS-MLD	Very low	Normal	Negative	-20q	Low
Patient 11	MDS-EB-2	Low	Normal	Negative	-17p	High
Patient 12	MDS-MLD	Very low	Normal	Negative	-17p	Low
Patient 13	MDS-EB-1	Intermediate	Normal	Negative	-17q	Very high
Patient 14	MDS-EB-2	Low	Normal	Negative	-19	Intermediate
Patient 19	MDS-MLD	High	Normal	Negative	-5q	Very high

*Negative just for -5/-5q, -7/-7q, +8, -20q, and 17p-

### Survival analysis

**Fig. 2:**
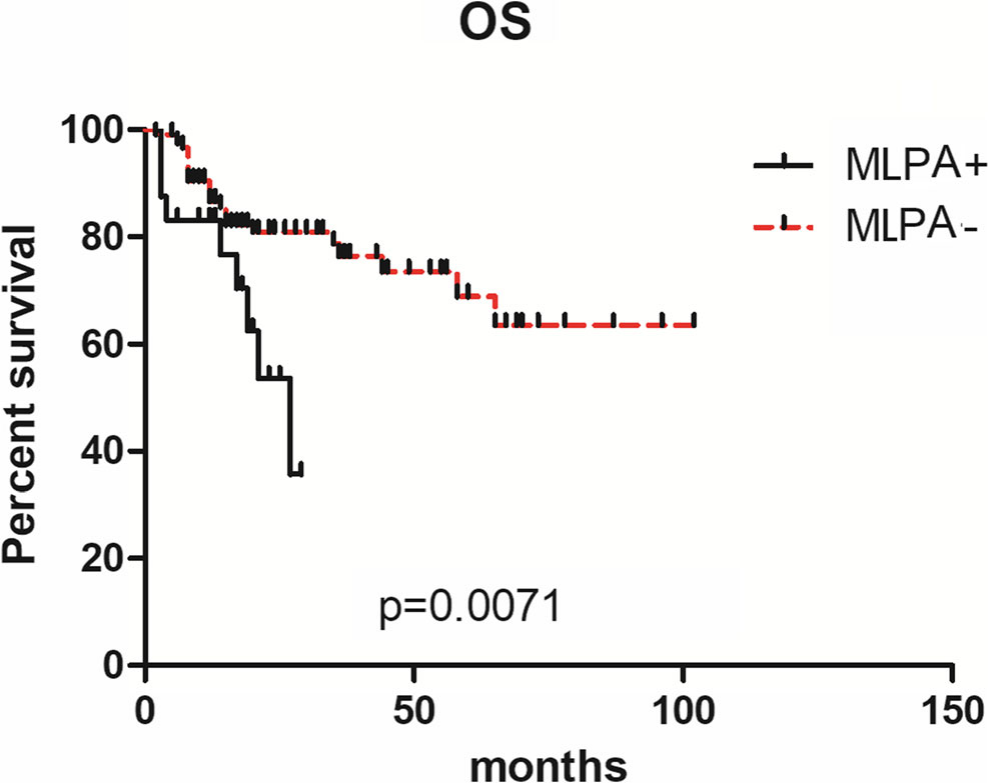
OS analysis of patients harboring aberration (n=24) or not detected (n=120 ) by MLPA in 144 MDS patients with normal and failed karyotype

We performed survival analysis and compared the outcome of patients which were also confirmed by MLPA (n=120) versus patients with aberrant karyotype as determined by MLPA (n=24). We observed a significant difference in survival (median OS: undefined vs 27 months, p=0.0071, Fig. 2). We performed a survival analysis of normal karyotypes and cases without result after banding cytogenetics, respectively. Data were shown in Supplement Fig.1.We can see that there was no significant difference (p=0.1877, p=0.2864) in the impact of MLPA results on OS of patients with normal karyotype and patients with failed karyotype. However, the curves of the two groups were clearly separated, and perhaps the difference was significant with the increase in the number of patients enrolled.

**Fig. 3:**
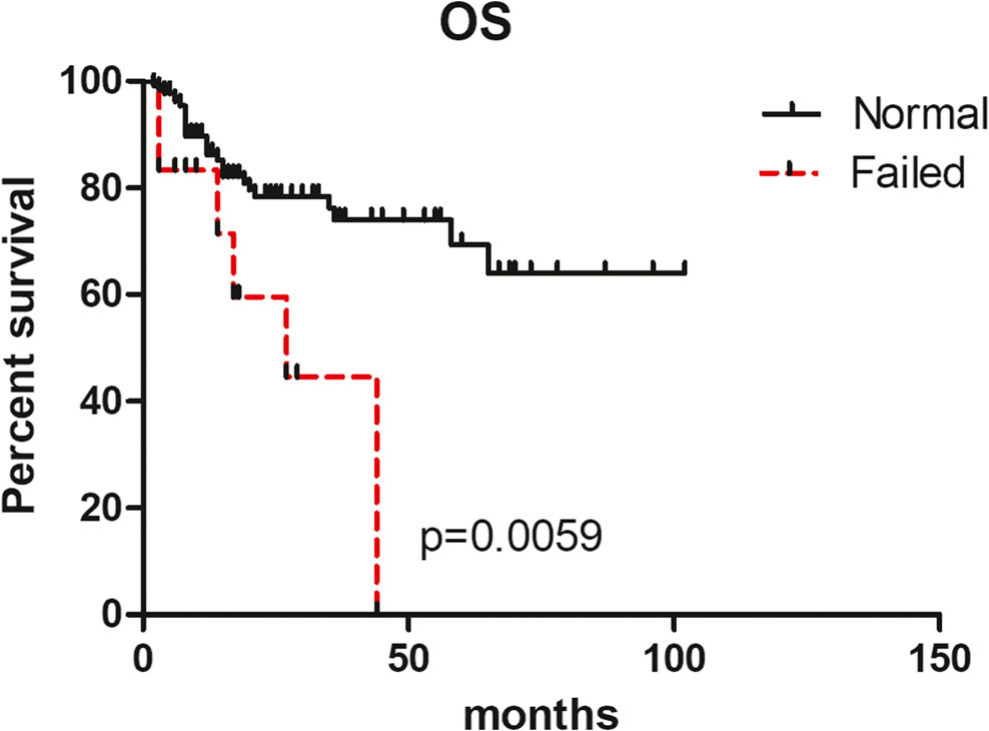
OS of patients with normal karyotype (n=132) and failed chromosome banding analysis (n=12)

In addition, we compared the outcome of patients with normal karyotype (n=132) to patients without result after banding cytogenetics (n=12). It showed a significant difference in survival (median OS: undefined vs 26 months, p=0.0059, Fig. 3), indicating that patients without result after banding cytogenetics had worse survival.

**Fig. 4:**
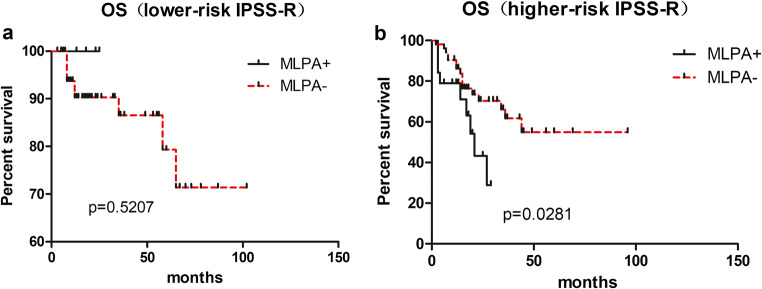
OS of patients with cytogenetic aberrations detected using MLPA compared with other patients in lower risk IPSS-R group (**a** MLPA+:n=5, MLPA-:n=68) and higher risk IPSS-R group (**b** MLPA+:n=19, MLPA-:n=52)

In our study, we also explored the impact of cytogenetic aberrations detected by MLPA on OS of lower-risk and higher-risk patients (defined according to IPSS-R) with a normal or without result after banding cytogenetics via R-banding test. Lower-risk IPSS-R group included very low-risk patients, low-risk patients, and intermediate patients with score≤3.5. Higher-risk IPSS-R group included intermediate patients with score >3.5, high risk, and very high-risk patients. For lower-risk IPSS-R patients (73/144), there were no differences in OS (p=0.5207; Fig. 4a). For higher-risk IPSS-R patients (71/144), OS was significantly shorter in the higher-risk patients with cytogenetic aberrations detected using MLPA (n=19) compared with other higher-risk patients (n=52) (median OS: 21 vs. undefined months, p=0.0281; Fig. 4b).

## Discussion

In MDS, the IPSS-R classifiers have clearly showed the prognostic impact of distinct cytogenetic abnormalities; these cytogenetic abnormalities include -7/7q-, -5/5q-, +8, 20q-, -Y, i(17q) or t(17p), -13/13q-, 11q-, 12p-, or t(12p), and the most common abnormalities (-7/7q-, -5/5q-, +8, and 20q-) occur in approximately 40% of MDS cases [10, 11]. The majority of chromosome aberrations in MDS are gains and deletions of chromosomal material, while balanced rearrangements are rare [3]. As we all know, more than 50% of MDS showed a normal karyotype. For MDS patients with normal karyotype, FISH is increasingly used for cytogenetic analysis because of its higher resolution and greater success rate. Nevertheless, it is difficult for FISH to screen all lesions simultaneously due to the high cost and technique limitation. Although established as the golden standard for detection of chromosomal aberrations in MDS, karyotyping (which requires cell amplification) and FISH are low-resolution, time-consuming, labor-intensive, and costly assays compared to MLPA, while small probes are directed at regions of interest in MDS, providing a resolution higher than that of FISH and bacterial artificial chromosome (BAC)-based array comparative genomic hybridization (aCGH) and equivalent to oligo-based aCGH [6, 7]. MLPA has a considerably higher resolution and can identify small unbalanced chromosomal aberrations undetectable by chromosome banding analysis.

Donahue et al. [12] showed that MLPA has higher accuracy and specificity than FISH in MDS and ALL patients. Array CGH and MLPA have been used as a method of choice for diagnosis of MDS patients with unexplained genetic aberrations. Volkert et al. [13] detected CNVs in 11% of 520 MDS patients with a normal karyotype using array CGH. Wang et al. [14] analyzed 437 MDS patients using an MLPA assay and detected clonal genetic abnormalities in 9.2% of cases with a normal or failed karyotype. In our study, we analyzed 258 MDS patients using MLPA assay and detected clonal genetic abnormalities in 16.7% of normal or failed karyotype patients. In our cohort, the proportion of patients with CNVs was higher, probably because of the higher proportion of patients with karyotype failure. For normal karyotype, MLPA detected clonal genetic abnormalities in 10.6% of 132 patients. Among 144 MDS patients, the consistency of FISH and MLPA was 95.1%, and no patient showed cytogenetic abnormalities detected just by FISH. Our results provide evidence that MLPA has an advantage over FISH for MDS patients.

As we all know, chromosome banding analysis is very important for MDS. Unfortunately, a variety of issues hamper cytogenetic evaluation in cases because chromosome banding studies may be hindered by several factors, including low proliferative rate in tissue culture, insufficient number of metaphase cells, reduced cell viability or hypo cellularity upon arrival to the reference laboratory, poor chromosome morphology, or complexity of the karyotype[15, 16]. So the outcomes of these patients were poorly understood. In our study, 4.6% of MDS patients showed  failed banding  cytogenetics, while we indicated that these patients may encounter a poor outcome as detected by MLPA (Fig. 3). On the other hand, the significance of failed banding cytogenetics on outcome of MDS patients has been scarcely reported. Medeiros et al. [17] compared the baseline characteristics and the prognostic impact of 94 (6%) AML patients with failed banding cytogenetics to the remaining 1403 AML patients with successful karyotype. These patients without result after banding cytogenetics had a lower response rate to induction chemotherapy, and the complete remission and survival rates were similar to those seen in patients with unfavorable karyotype. Lazarevic et al. [18] analyzed 1737 AML patients; the frequencies of unsuccessful cytogenetics and unperformed cytogenetics were 2.1% and 20%, respectively. Their research showed that a lack of cytogenetic data translates into a poor prognosis. Our findings support this observation that patients with failed karyotype should be considered higher-risk patients. Together, new techniques such as MLPA should be used to overcome the technical challenges associated with cases without result after banding cytogenetics.

For the impact of cytogenetic aberrations detected by MLPA, as shown in Fig. 2, OS in 24 patients harboring abnormalities detected just by MLPA was significantly shorter compared to others. The impact of cytogenetic aberrations detected by MLPA on OS of different IPSS-R patients is also shown in Fig. 4. These results are consistent with the study of Wang [16]. All those suggest that MLPA has the potential to alter the risk stratification in MDS patients with normal or cases without result after banding cytogenetics.

## Conclusion

In conclusion, MLPA can detect CNVs in a high-throughput fashion with higher resolution and can be used easily in routine diagnostics in MDS with normal karyotype or cases without result after banding cytogenetics, benefiting to the patients harboring submicroscopic deletions where informative prognostic factors underlying.

## Supplementary information

Supplementary Fig.1 a. OS analysis of patients harboring aberration (n=14 ) and not detected (n=118)by MLPA in 132 MDS patients with normal karyotype. b. OS analysis of patients harboring aberration (n=10) and not detected (n=2) by MLPA in 12 MDS patients with failed karyotype. (PNG 79 kb)

High resolution image (TIF 812 kb)
