# Supplementary figures and images for: Multiplex ligation-dependent probe amplification identifies copy number changes in normal and undetectable karyotype MDS patients
Source: Ann Hematol. 2021 May 15;100(9):2207–14. doi: 10.1007/s00277-021-04550-8 (PMC8357724; doi:10.1007/s00277-021-04550-8)

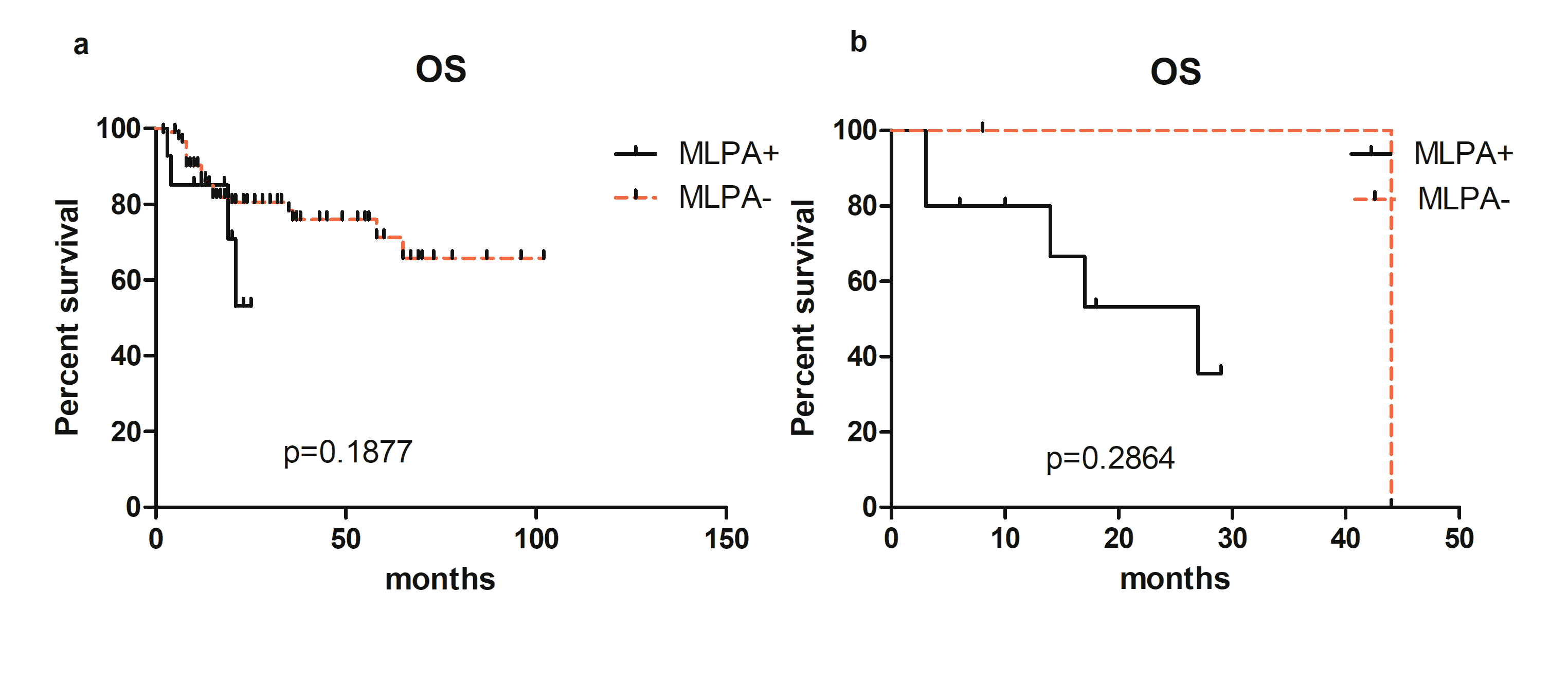

Supplement: Supplementary file 1 — a. OS analysis of patients harboring aberration (n=14 ) and not detected (n=118)by MLPA in 132 MDS patients with normal karyotype. b. OS analysis of patients harboring aberration (n=10) and not detected (n=2) by MLPA in 12 MDS patients with failed karyotype. (PNG 79 kb) [file 277_2021_4550_Fig5_ESM.png]

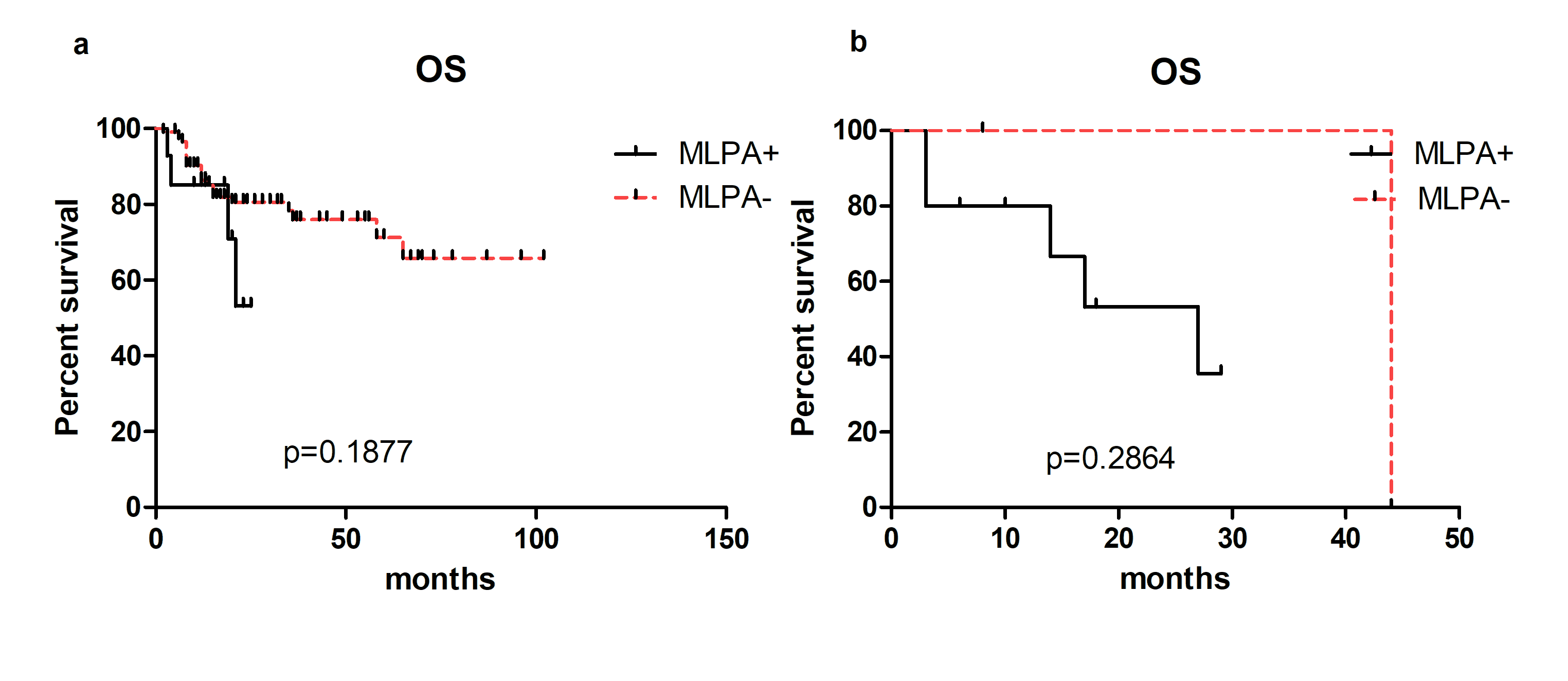

Supplement: Supplementary file 2 — High resolution image (TIF 812 kb) [file 277_2021_4550_MOESM1_ESM.tif]
